# O.3.2-3 ‘It helped me to disconnect from the problems’: a qualitative study exploring the subjective experiences of people with schizophrenia in a concurrent exercise program

**DOI:** 10.1093/eurpub/ckad133.148

**Published:** 2023-09-11

**Authors:** Mikel Tous-Espelosin, Diane Crone, Nagore Iriarte-Yoller, Sara Maldonado-Martin

**Affiliations:** GIzartea, Kirola eta Ariketa Fisikoa Ikerkuntza Taldea (GIKAFIT), Society, Sports, and Physical Exercise Research Group, Department of Physical Education and Sport, Faculty of Education and Sport-Physical Activity and Sport Sciences Section, University of the Basque Country (UPV/EHU), Vitoria-Gasteiz, Araba/Álava, Basque Country, Spain; Bioaraba Health Research Institute, Physical Activity, Exercise, and Health Group, Vitoria-Gasteiz, Basque Country, Spain; Centre for Health, Activity and Wellbeing Research, Cardiff Metropolitan University, Cardiff CF236XD, UK; Bioaraba, New Therapies in Mental Health Group, Vitoria-Gasteiz, Spain; Osakidetza Basque Health Service, Araba Mental Health Network, Psychiatric Hospital of Alava, Vitoria-Gasteiz. Spain; Department of Medicine, Faculty of Health Sciences, University of Deusto, Bilbao, Spain; mikel.tous@ehu.eus; GIzartea, Kirola eta Ariketa Fisikoa Ikerkuntza Taldea (GIKAFIT), Society, Sports, and Physical Exercise Research Group, Department of Physical Education and Sport, Faculty of Education and Sport-Physical Activity and Sport Sciences Section, University of the Basque Country (UPV/EHU), Vitoria-Gasteiz, Araba/Álava, Basque Country, Spain; Bioaraba Health Research Institute, Physical Activity, Exercise, and Health Group, Vitoria-Gasteiz, Basque Country, Spain

## Abstract

**Purpose:**

The relationship between exercise and its role in mental health is extensively documented and has led to the inclusion of exercise into adjuvant programs for people with mental health disorders. However, considerably less attention is devoted to the experience of taking part in exercise and perceived health outcomes for people with specific mental health disorders. The aim of this study was therefore to investigate the subjective experiences of a concurrent exercise program designed to improve both physical and mental health, through participation, for people with schizophrenia.

**Methods:**

Participants diagnosed with schizophrenia (n = 35, 41.6±10.3 years) from two locations (a psychiatric hospital and a mental health community support group) received an intensive concurrent exercise program (*i.e.,* aerobic interval training and resistance training within the same session) for a five-month duration, three times a week, at out-of-hospital facilities. Qualitative data was collected via individual, semi-structured interviews, organized, and analyzed with thematic analysis using NVivo software.

**Results:**

A conceptual model developed from the analysis explains the participants’ subjective experiences, opinions, and perceptions of the role of exercise and their perceived outcomes from participation in the program. Six themes emerged from the analysis. The themes ‘expectations about taking part’ and ‘attitude to exercise, the program, and general lifestyle’ appeared to influence participants to take part. The act (and factors associated with it) of ‘taking part’ then led to subsequent ‘perceived outcomes’ and ‘plans’.

**Conclusions:**

Findings from the study support the case for the strategic use of exercise as an adjuvant program and maintenance of holistic health for people with schizophrenia. However, this study also demonstrates the paramount importance of building a continuation plan for participation to ensure sustainable and long-term adherence to exercise. The location of the program appears important to consider (*i.e.,* out-of-hospital), as does the constitution of the group, the type of people, and the exercise program itself. For this clinical group, the exercise intervention not only serves a physical and mental enhancement function but also appears to achieve holistic health benefits such as well-being and important opportunities for social interaction.

